# A novel linear and broadly neutralizing peptide in the SARS-CoV-2 S2 protein for universal vaccine development

**DOI:** 10.1038/s41423-021-00778-6

**Published:** 2021-10-13

**Authors:** Tuofan Li, Qiuqi Kan, Jinying Ge, Zhimin Wan, Mengqi Yuan, Yao Huang, Quan Xie, Yi Yang, Hongxia Shao, Xiangdong Li, Lilin Ye, Aijian Qin, Zhigao Bu, Pinghuang Liu, Jianqiang Ye

**Affiliations:** 1 Jiangsu Coinnovation Center for Prevention and Control of Important Animal Infectious Diseases and Zoonoses, Yangzhou University, Yangzhou, 225009 Jiangsu China; 2 Key Laboratory of Jiangsu Preventive Veterinary Medicine, Key Laboratory for Avian Preventive Medicine, Ministry of Education, College of Veterinary Medicine, Yangzhou University, Yangzhou, 225009 Jiangsu China; 3 Joint International Research Laboratory of Agriculture and Agri-Product Safety, the Ministry of Education of China, Yangzhou University, Yangzhou, 225009 Jiangsu China; 4 Institutes of Agricultural Science and Technology Development, Yangzhou University, Yangzhou, 225009 Jiangsu China; 5 State Key Laboratory of Veterinary Biotechnology, Harbin Veterinary Research Institute, Chinese Academy of Agricultural Sciences, 150069 Harbin, China; 6 National High Containment Laboratory for Animal Diseases Control and Prevention, 150069 Harbin, China; 7 College of Veterinary Medicine, China Agricultural University, 100193 Beijing, China; 8 Center of Diseases Control and Prevention, Yangzhou, 225009 Jiangsu China; 9 Institute of Immunology, PLA, Third Military Medical University, 400038 Chongqing, China

**Keywords:** Infectious diseases, Vaccines, Immunotherapy

As humans continue to develop COVID-19 widely, numerous novel variants of SARS-CoV-2 have emerged [1, 2]. These variants, which may possess enhanced transmissibility and often result in breakthrough infections in the vaccinated population, pose great challenges to the current vaccine strategies targeting the immunodominance of the receptor-binding domain (RBD) of the spike (S) protein [2].

Similar to SARS-CoV, SARS-CoV-2 uses angiotensin-converting enzyme 2 (ACE2) as the receptor for binding to host cells [3]. During infection, the S protein of SARS-CoV-2 is first cleaved into S1 and S2 subunits; the RBD of the S1 subunit binds to ACE2, and the S2 subunit mediates viral fusion with the cell membrane [3]. In contrast to S1, which is prone to frequent mutation, S2 is highly conserved and can serve as an attractive target for broad protection [4]. However, protective epitopes in the S2 subunit have not yet been extensively characterized [5, 6].

In our effort to develop universal SARS-CoV-2 vaccine candidates, four peptides (Table S1) derived from the S protein were synthesized for systematic characterization. In an enzyme-linked immunosorbent assay (ELISA), 11 of 19 serum samples collected from recovered COVID-19 patients recognized the P4 peptide but not the other three (Fig. 1a), indicating that P4 carries potent B cell epitopes.

**Fig. 1:**
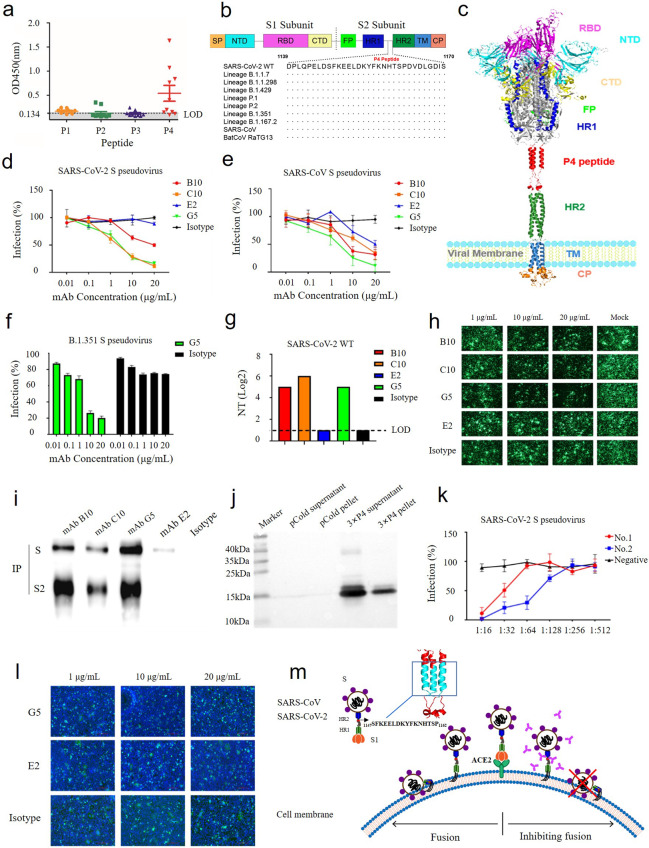
A novel linear and broadly neutralizing peptide in the S2 protein of SARS-CoV-2. **a** ELISA with the indicated peptides as antigens was used to test sera from recovered COVID-19 patients. LOD, limit of detection. **b** Sequence alignment showing the location of P4 in SARS-CoV-2, SARS-CoV, BatCoV RaTG13 and SARS-CoV-2 variants. **c** A 3D structure model diagram of the SARS-CoV-2 S protein generated with PyMOL based on PDB 6VSB_1_1_1 [10]. The P4 peptide is shown in red. **d** and **e** Neutralizing activity of B10, C10, E2, and G5 mAbs against SARS-CoV-2 S pseudovirus (**d**) and SARS-CoV S pseudovirus (**e**). **f** Neutralizing activity of mAb G5 against the SARS-CoV-2 S pseudovirus B.1.351 South Africa variant. In (**d**–**f**), measurements were performed using purified mAbs and SARS-CoV-2 and SARS-CoV S pseudoviruses in hACE2/293T cells, and the results are shown as the percentage of inhibition (mean ± SD). **g** Neutralizing activity of B10, C10, E2, and G5 mAbs against wild-type SARS-CoV-2. The assay was performed with mouse ascites and wild-type SARS-CoV-2 in Vero E6 cells, and the data are expressed as the log2 number of dilution. **h** Inhibition of S protein-mediated virus spread by mAbs. **i** Affinities of B10, C10, E2, and G5 mAbs for the S protein were analyzed by immunoprecipitation assays. 293T cells were transfected with a plasmid carrying the SARS-CoV-2 S gene, and mAbs B10, C10, E2, and G5 were used as the capture antibody to perform immunoprecipitation, followed by western blot analysis using mAb G5. **j** Identification of the trimeric P4 protein by western blotting. The supernatants and pellets from lysates of BL21 cells transformed with 3×P4 or the pColdI vector were analyzed by western blotting using mAb G5 as the primary antibody. **k** Neutralization of SARS-CoV-2 pseudovirus by sera from mice immunized with the trimeric P4 protein. The assay was performed with mouse sera and SARS-CoV-2 pseudovirus in hACE2/293T cells. **l** Inhibition of S-mediated cell-cell fusion by mAbs. **m** A schematic diagram showing the potential mechanism by which mAbs B10, C10, E2, and G5 neutralize SARS-CoV-2/SARS-CoV. mAbs against _1147_SFKEELDKYFKNHTSP_1162_ inhibit SARS-CoV-2/SARS-CoV viral entry by inhibiting fusion between the virus and cell membrane

The P4 peptide of the S2 subunit, with the amino acid sequence DPLQPELDSFKEELDKYFKNHTSPDVDLGDIS (corresponding to residues 1139-1170 of the S protein), is located in the linker region between heptad repeat 1 (HR1) and heptad repeat 2 (HR2). The P4 peptide is highly conserved among SARS-CoV, BatCoV RaTG13, SARS-CoV-2 and the recent SARS-CoV-2 variants, including those from lineages B.1.1.7, B.1.1.298, B.1.429, P.2, P.1, B.1.351, and B.1.617.2 (Fig. 1b, c). Because HR1 and HR2 play critical roles in mediating the fusion process of coronavirus [7], we hypothesize that the P4 peptide is a potential target for developing peptide-based inhibitors and vaccines. To identify potential B cell epitopes in P4, we immunized BALB/c mice with the P4 peptide conjugated with KLH (P4-KLH) and generated four monoclonal antibodies (mAbs), designated B10, C10, E2, and G5. These mAbs bound to the S and S2 proteins of both SARS-CoV-2 and SARS-CoV, as revealed by immunofluorescence assays and western blotting (Fig. S1a-c), confirming their specificity for the S2 protein.

To examine neutralization, these mAbs were tested in assays using hACE2/293T cells. mAbs B10, C10, and G5 efficiently neutralized SARS-CoV-2 S-pseudovirus, with half maximal inhibitory concentrations (IC_50_) of 18.05, 1.367, and 2.21 μg/mL, respectively (Fig. 1d). Conversely, mAb E2 did not show detectable neutralization of SARS-CoV-2 S-pseudovirus. mAbs B10, C10, and G5 also effectively neutralized SARS-CoV S-pseudovirus, with IC_50_ values of 5.295, 2.325, and 2.695 μg/mL (Fig. 1e). Antibody G5 neutralized a SARS-CoV-2 pseudovirus bearing the S protein from the B.1.351 variant lineage, with an IC_50_ of 2.608 μg/mL (Fig. 1f). In line with these data, mAbs B10, C10, and G5 (tested with mouse ascites containing mAbs) were able to neutralize wild-type SARS-CoV-2 (Fig. 1g). Moreover, mAbs B10, C10, and G5, but not mAb E2 and the control mAb, inhibited the spread of a vesicular stomatitis virus expressing SARS-CoV-2 S and green fluorescent proteins (VSV-SARS-CoV2-S-GFP) in Vero E6 cells (Fig. 1h). Moreover, immunoprecipitation assays revealed that mAbs B10, C10, and G5 efficiently immunoprecipitated both the S and S2 proteins of SARS-CoV-2 but that mAb E2 only immunoprecipitated the S protein of SARS-CoV-2 at low efficiency (Fig. 1i).

Western blotting using truncated P4 demonstrated that all the mAbs reacted with only S2-P4 and S-P4-1 and not the other truncations (Table S2 and Fig. S1d,e), which indicates that the epitopes recognized by these mAbs are located in _1147_SFKEELDKYFKNHTSP_1162_ in S2 and that the eight amino acids in _1155_YFKNHTSP_1162_ are critical for the epitopes. Whether these mAbs recognize the same epitope or different epitopes needs to be further elucidated. To evaluate whether the epitope in the P4 peptide is of value for vaccine development, a trimeric P4 expression vector, 3×P4, was constructed for protein expression (Table S3 and Fig. S2a, b). Trimeric P4 was readily recognized by mAb G5, highlighting the antigenicity of the expressed trimeric P4 protein (Fig. 1j). In addition, sera from mice immunized with the purified trimeric P4 protein neutralized SARS-CoV-2 S-pseudovirus in a dose-dependent manner (Fig. 1k). These data demonstrate that trimeric P4 is a potential vaccine candidate for broad protection against SARS-CoV-2.

To further investigate the mechanism underlying inhibition of SARS-CoV-2 by these mAbs, we performed a cell-cell fusion inhibition assay, as previously described [7]. mAb G5, but not E2 or the control mAb, inhibited SARS-CoV-2-S-mediated cell-cell fusion in a dose-dependent manner (Fig. 1l). Thus, antibodies against P4 might inhibit viral entry by blocking fusion of the virus with the host cell membrane.

In conclusion, we identified a novel linear and broadly neutralizing peptide in the S2 protein of SARS-CoV-2. Different from other cross-neutralizing epitopes [4–9], this linear peptide (_1147_SFKEELDKYFKNHTSP_1162_) is conserved across SARS-CoV, BatCoV RaTG13, SARS-CoV-2, and SARS-CoV-2 variants. mAbs targeting this peptide efficiently neutralized SARS-CoV-2 and SARS-CoV S-pseudovirus as well as wild-type SARS-CoV-2. These antibodies also inhibited both S-mediated cell-cell membrane fusion and viral spread among cells. Furthermore, the recombinant trimeric P4 carrying the _1147_SFKEELDKYFKNHTSP_1162_ epitope induced neutralizing antibodies against SARS-CoV-2 S-pseudovirus. Taken together, these findings suggest that antibodies targeting the peptide _1147_SFKEELDKYFKNHTSP_1162_ may neutralize both SARS-CoV-2 and SARS-CoV by preventing fusion between the virus and cell membrane (Fig. 1m). In light of the rapid emergence of antigenic variants of SARS-CoV-2, the identification of neutralizing peptides such as _1147_SFKEELDKYFKNHTSP_1162_ represents an opportunity for the development of universal vaccines and therapeutic reagents against current pandemic strains as well as future SARS-CoV-2 mutants.

## Supplementary information

Additional information

## Data Availability

The datasets used and/or analyzed during the current study are available from the corresponding author on reasonable request.
