# Supplementary material for: A novel linear and broadly neutralizing peptide in the SARS-CoV-2 S2 protein for universal vaccine development
Source: Cell Mol Immunol. 2021 Oct 13;18(11):2563–5. doi: 10.1038/s41423-021-00778-6 (PMC8513545; doi:10.1038/s41423-021-00778-6)
Supplement: Supplementary file 1 — Additional information [file 41423_2021_778_MOESM1_ESM.docx]

**Additional information**

**Materials and Methods**

**Cells and plasmids**

HEK293T cells were preserved in our laboratory and cultured in Dulbecco’s modified Eagle’s medium (DMEM) with 5% fetal bovine serum (FBS) and Vero E6 cells were cultured in DMEM with 10% FBS at 37℃ with 5% CO_2_. The hACE2-expressing HEK-293T (hACE2/293T) cells were cultured in DMEM with 10% FBS and 500 μg/mL G418 as described elsewhere.^1^ The pcDNA3.1-SARS-CoV-2-S and pCMV3-Spike(SARS-CoV)-FLAG plasmids with human codon optimization were purchased from HedgehogBio (Shanghai, China) and MiaoLing Plasmid Sharing Platform (Wuhan, China), respectively.

**Peptide-based indirect ELISA for screening antibodies against SARS-CoV-2**

All the peptides used in this study were synthesized by Dechibio (Shanghai, China) with a purity of >90% (tested by high-performance liquid chromatography). The amino acid sequence of P1, P2, P3 and P4 were shown in Table 1. P1, P2, P3 and P4 peptides diluted in 0.1 M carbonate/bicarbonate buffer (pH 9.6) were respectively used to coat 96-well plates (0.5 μg/well) at 4℃ overnight. The ELISA plates were washed with PBST (0.01 M phosphate-buffered saline (PBS), pH 7.2, 0.05% Tween 20) once and then blocked with PBST containing 5% skim milk and 1% fetal bovine serum for 1 h at 37℃. After washed once with PBST, the SARS-CoV-2 patient sera or the supernatant from the hybridoma cells or of the diluted mAbs were added and incubated for 1 h at 37 °C. After three washes with PBST, 100 μL of a 1:10000 dilution of HRP-labelled goat anti-mouse IgG was added into the plates with. After incubated for 1 h at 37 °C, the ELISA plates were washed three times with PBST, and then 100 μL TMB was added (Solarbio, Beijing, China) for development. After 8 min at 37 °C, the development reaction was stopped by adding 50 μL of 2 M H_2_SO_4_, and the absorbance values at 450 nm (OD450) were measured with a Microplate Reader (TECAN Infinite M200 Pro). To determine the cut-off value of the ELISA, 64 SARS-CoV-2 negative human sera samples were tested by the ELISA. This cut-off was determined according to the OD450 by calculating the arithmetic mean plus three times of the standard deviation. When the OD450 value of the sample was higher than 0.134, the sample was regarded as positive.

**Generation of mAbs**

Fifty micrograms of P4-KLH were used to immunize 6-week-old BALB/c mice four times every 10 days. At day 3 following the fourth immunization, the splenic cells from one immunized mouse were collected and fused with sp2/0 cells with PEG2000 as previously described.^2^ After cultured with HAT-selective medium (Sigma), the positive [hybridoma](https://www.sciencedirect.com/topics/immunology-and-microbiology/hybridoma) were screened using 293T cells transfected with SASR-CoV-2 S plasmid by immunofluorescence assay (IFA). After sub-cloning of the positive hybridoma cells, the characteristics of the mAbs were identified by IFA and western blot using SASR-CoV-2 S protein as antigen, and by P4-peptide ELISA. The isotype of mAb was determined with a mouse mAb isotyping kit (Thermo Scientific, Massachusetts, USA) according to the manufacturer’s protocol. The mAbs in ascites were generated as previously described and purified using protein G columns (GE Healthcare Life sciences, Uppsala, Sweden).^3^

**Immunofluorescence assay (IFA)**

293T cells were transfected with pcDNA3.1-SARS-CoV-2-S, pCMV3-Spike (SARS-CoV)-FLAG and pcDNA3.1 empty vector, respectively. Two days post transfection (dpt), the cells were fixed with 4% paraformaldehyde in PBS for 15 min and then treated with 0.25% Triton-X100 for 10 min at room temperature (RT). Then, the cells were blocked using 1% bovine serum albumin (BSA) for 30 min at 37 °C. The mAbs (1:500 dilution in PBS with 1% BSA) were incubated with the fixed cells for 45 min at 37 °C. After three washes with PBS, FITC-conjugated secondary antibodies at 1:150 dilutions were incubated with the cells for another 45 min at 37 °C. After three washes with PBS, the plates were observed with an inverted fluorescence microscope.

**Immunoblotting**

The transfected cells were collected and lysed in lysis buffer. The lysates were collected after centrifugation for 20 min at 12000 rpm and the supernatants were boiled with loading buffer and analyzed by western blot. After blocking with PBST containing 5% skim milk for 2 h at RT, the nitrocellulose membranes (NCs) (GE Healthcare Life sciences, Freiburg, Germany) were incubated with ascites (1:1000 dilution in PBST) for 2 h at RT. After three washes with PBST, the NCs were incubated with HRP-conjugated goat anti-mouse IgG (1:15000 dilution in PBST) for 1 h at RT. After three washes, the NCs were developed using a fully automatic chemiluminescence image analysis system (Tanon 5200).

**Pseudovirus neutralization assay**

Spike protein of SARS-CoV-2 or SARS-CoV typed pseudovirus expressing Luciferase (S-pseudovirus) was purchased from Novobiosci (Shanghai, China). The pseudovirus neutralization assay was performed as previously described with some modification.^1^ In brief, the mAbs or polyclonal antibodies were diluted and mixed with SARS-CoV-2 or SARS-CoV pseudovirion. The mixture was further incubated on ice for 1 h and then added into hACE2-expressing HEK-293T (hACE2/293T) cells for overnight incubation. On day 2, the cells were washed once with PBS and fresh medium was supplied. At 60 hours-post incubation, the luciferase activity was measured using the Dual Luciferase Reporter Assay Kit (Vazyme, Nanjing, China) as the manufacturer’s protocol described.

**Inhibition of S protein-mediated virus diffusion by mAbs**

To determine whether the mAb could inhibit the virus diffusion mediated by SARS-CoV-2 S protein, VSVΔG-S-GFP virus was used to test in Vero-E6 cells. In brief, Vero-E6 cells were inoculated into 24-well plate. Next day, VSVΔG-S-GFP virus was incubated with the cells at an MOI of 0.1 for 1 h. Then, the cells were washed with DMEM for three times and culture in 10% FBS DMEM supplied mAb at the indicated concentrations. Cells were cultured for 24 h and the GFP-signal was observed and captured by an inverted fluorescence microscope.

**Authentic SARS-CoV-2 neutralization assay**

The two-fold serially diluted mAbs were respectively mixed with authentic SARS-CoV-2 (200 TCID_50_) and further incubated at 37℃ for 1 h. Then, the mixture was added into Vero E6 cells in 96-well plate and incubated for another 1 h. And 50 μL fresh medium was then supplied to the cells and the cells were cultured for 72 hours post infection. The neutralization assay result was determined by detecting the viral plaques. The neutralization titer was indicated using the highest serum dilution that inhibited at least half of the plaques. The authentic SARS-CoV-2 assays were performed in the biosafety level 4 facilities in the Harbin Veterinary Research Institute (HVRI) of the Chinese Academy of Agricultural Sciences (CAAS), which is approved for such use by the Ministry of Agriculture and Rural Affairs of China.

**Immunoprecipitation**

One microgram of the mAbs was respectively mixed with 40 μL protein G-Sepharose beads (Beyotime) at 4℃ for 3 h. The mAb-beads mixtures were washed three times with PBS and further mixed with the lysate of 293T cells transfected with pc- SARS-CoV-2-S at 4℃ for another 3 h. Then, the mixtures of proteins and beads were washed five times with the cold PBS, and the immunoprecipitated proteins were boiled with the loading buffer and analyzed by western blot.

**Epitope mapping**

To map the epitopes that the mAbs recognized, serial SARS-CoV-2-S2 recombinant constructs with flag tag and various deletions at the C-terminus were generated with a ClonExpress II One Step Cloning Kit (Vazyme Biotech, Nanjing, China) as previously described.^4^ The primers used for amplifying the SARS-CoV-2 S2 gene with different deletions and the linear pcDNA3.1 vector are listed in Table 2. 293T cells transfected with the different SARS-CoV-2-S2 recombinants were lysed at 2 dpt and analyzed using the indicated mAbs via western blot, as described above.

**Generation of the trimeric P4 protein and its polyclonal antibody in mice**

To generate the trimeric P4 protein, three DNA segments of P4 with linker (amino sequence: GGGGS) between each other were cloned into the prokaryotic expression vector pColdⅠ vector for obtaining immunogen. In brief, three fragments of P4 with different homologous arms were amplified using pcDNA3.1-SARS-CoV-2-S as template with the primers shown in Table 3. Then, the three purified fragments were mixed with linearized pCold Ⅰ vector and ligated via homologous recombination using a ClonExpress® MultiS One Step Cloning Kit (Vazyme, Nanjing, China). The diagrammatic sketch of the generated recombinant plasmid was shown in Fig. 4a and designated as pCold Ⅰ-3×P4. The pCold Ⅰ-3×P4 plasmid was transformed to BL21 (DE3) component cells, and induced using IPTG overnight at 16℃. The 3×P4 protein with His tag was purified from the supernatant of the lysed bacteria via His-tag affinity chromatography (GE Healthcare Life sciences, Uppsala, Sweden). The purified trimeric P4 protein (50 μg/mouse) was used to immunize 6-week-old BALB/c mice five times every 20 days, and the mouse sera against the trimeric P4 were isolated at day 20 post the fifth immunization.

**Inhibition of S-mediated cell-cell fusion by mAbs**

The establishment and detection of SARS-CoV-2 S-mediated cell-cell fusion assay was performed as previously described,^5^ with some modifications. 293T/ACE2 cells were used as target cells and the 293T cells transfected with pcDNA3.1-SARS-CoV-2-S-EGFP plasmid were used as effector cells. The inhibitory effect of the mAbs on SARS-CoV-2 S-mediated cell-cell fusion was evaluated as previously described. Briefly, to prepare effector cells expressing SARS-CoV-2 S protein, 293T cells were transfected with pcDNA3.1-SARS-CoV-2-S-EGFP or pcDNA3.1-EGFP for 48h. Afterward, 2×10^4^ cells/well of the target cells (293T/ACE2) were pre-incubated at 37℃ for 5 h. At 48 h post-transfection, the effector cells were harvested and 10^4^ cells/well of the effector cells were incubated with the mAbs at the indicated concentrations at 37℃ for 30 min. Then, the effector cells with mAbs were added into the target cells and further cultured for 24 h. Cells were fixed with 4% paraformaldehyde, treated with 0.25% Triton-X100 in PBS, blocked with 3% BSA in PBS and stained with hoechst 33342. The expression of SARS-CoV-2-S-EGFP was observed based on GFP fluorescent and the cell-cell fusion was monitored by larger but weaker GFP-signal with multi-nucleated syncytia compared to the unfused cells with normal nucleus. The images were captured using inverted fluorescence microscope (Nikon).

**References for additional information**

1. Chen, X. et al. Human monoclonal antibodies block the binding of SARS-CoV-2 spike protein to angiotensin converting enzyme 2 receptor. *Cell Mol. Immunol.* **17**, 647-649 (2020).

2. Nelson, P. N. et al. Monoclonal antibodies. *Mol. Pathol.* **53**, 111-7 (2000).

3. Ye, J. et al. Intranasal delivery of an IgA monoclonal antibody effective against sublethal H5N1 influenza virus infection in mice. *Clin. Vaccine Immunol.* **17**, 1363-70 (2010).

4. Wang, P. et al. A novel monoclonal antibody efficiently blocks the infection of serotype 4 fowl adenovirus by targeting fiber-2. *Vet. Res.* **49**, 29 (2018).

5. Xia, S. et al. Inhibition of SARS-CoV-2 (previously 2019-nCoV) infection by a highly potent pan-coronavirus fusion inhibitor targeting its spike protein that harbors a high capacity to mediate membrane fusion. *Cell Res.* **30**, 343-355 (2020).

**Table S1 The synthetic peptides in this study**

| Peptide | Amino acid sequence |
| --- | --- |
| P1 | _145_yhknnkswmesefrvyssannc_166_ |
| P2 | _404_gdevrqiapgqtgkiadynyklpddftgcvi_434_ |
| P3 | _797_pikdfggfnfsqilpdpskpskrsfiedl_821_ |
| P4 | _1139_dplqpeldsfkeeldkyfknhtspdvdlgdis_1170_ |

**Table S2 Primers for construction of SARS-CoV-2 S2 truncations**

|  | **Sequences of primers (5’-3’)** |
| --- | --- |
| **S2-P4** | F: AGCTTGGTACCGAATGTCTGTGGCCTCTCAGTCT |
|  | R: ATATCTGCAGAATTTACTTATCGTCGTCATCCTTGTAATCGCTAATGTCGCCCAGGTC |
| **S2-P4-1** | F: AGCTTGGTACCGAATGTCTGTGGCCTCTCAGTCT |
|  | R: ATATCTGCAGAATTTACTTATCGTCGTCATCCTTGTAATCGGGAGATGTGTGGTTCTT |
| **S2-P4-2** | F: AGCTTGGTACCGAATGTCTGTGGCCTCTCAGTCT |
|  | R: ATATCTGCAGAATTTACTTATCGTCGTCATCCTTGTAATCCTTGTCAAGTTCCTCCTT |
| **S2-P4-3** | F: AGCTTGGTACCGAATGTCTGTGGCCTCTCAGTCT |
|  | R: ATATCTGCAGAATTTACTTATCGTCGTCATCCTTGTAATCGTCAAGTTCGGGTTGCAA |
| **Linear pcDNA3.1** | F: GAATTCTGCAGATATCCAGCACAGTG |
|  | R: GCTCGGTACCAAGCTTAAGTTTAAACG |

**Table S3 Primers for construction of pColdⅠ-3×P4 plasmid**

|  | **Sequences of primers (5’-3’)** |
| --- | --- |
| **N-P4** | F: ATGGAGCTCGGTACCGACCCATTGCAACCCGAACTTG |
|  | R: ACTTCCACCTCCACCGCTAATGTCGCCCAGGTCCAC |
| **Middle-P4** | F: GGTGGAGGTGGAAGTGACCCATTGCAACCCGAACTTG |
|  | R: ACTTCCACCTCCACCGCTAATGTCGCCCAGGTCCAC |
| **C-P4** | F: GGTGGAGGTGGAAGT GACCCATTGCAACCCGAACTTG |
|  | R: CAGGTCGACAAGCTTTCAGCTAATGTCGCCCAGGTCCAC |
| **Linear pCold Ⅰ** | F: AAGCTTGTCGACCTGCAGTCTAGAT |
|  | R: GGTACCGAGCTCCATATGCCTACC |

**Supplementary Figure legends**


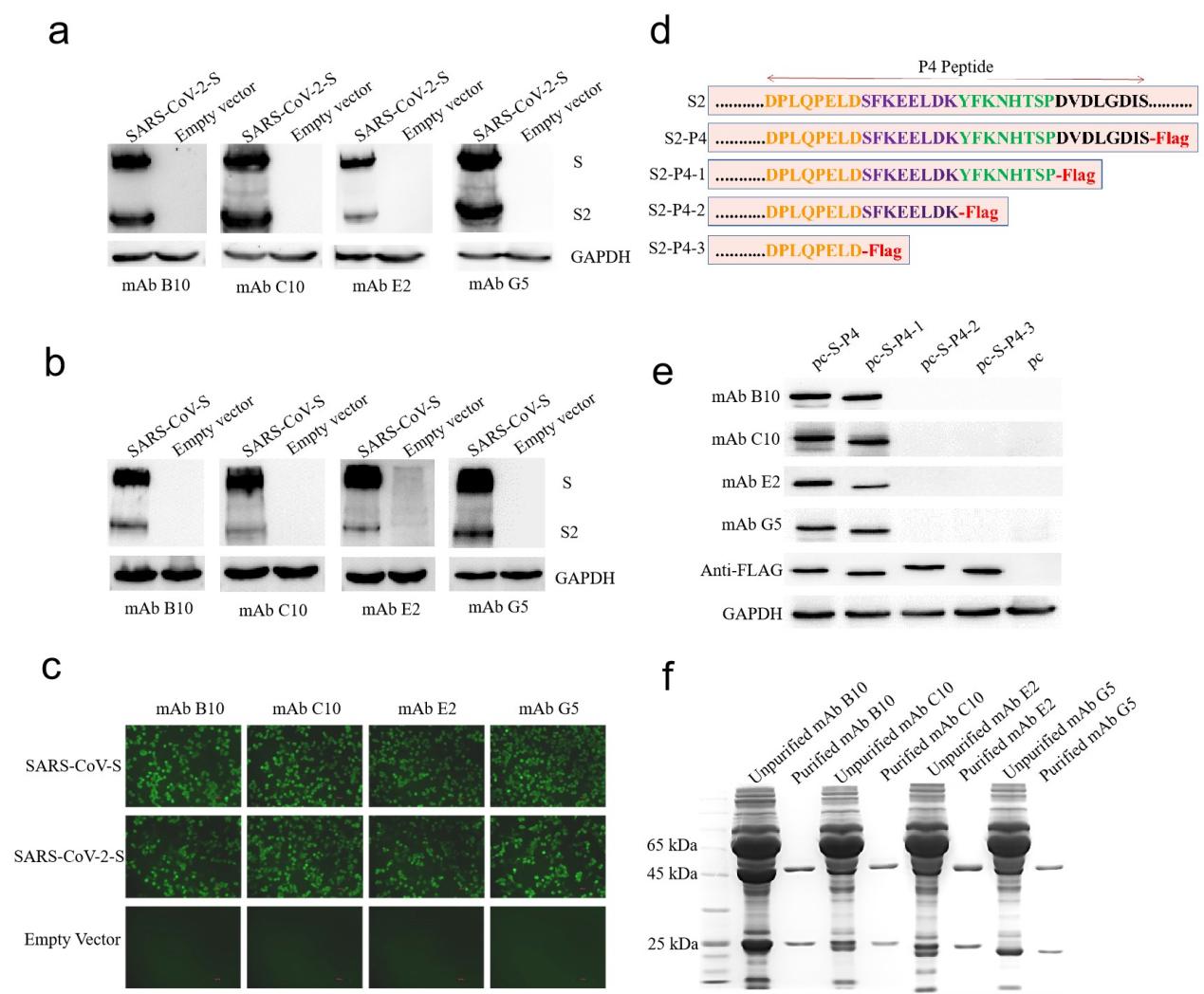


**Fig S1. mAbs against P4 recognized the linear S of SARS-CoV-2 and SARS-CoV.**

**a-c,** Specificity analysis for the mAbs by western blot and IFA. 293T cells were transfected with SARS-CoV2, SARS-CoV S and pcDNA3.1 empty vector, respectively. Then, mAbs of B10, C10, E2 and G5 were respectively used as the primary antibody to perform western blot (**a**, **b**) or IFA (**c**). **d, e,** Epitope mapping for the mAbs using the truncated SARS-CoV-2 S2. (**d**) Strategy for construction of the truncated SARS-CoV-2 S2 constructs. The C terminus part of the sequence of constructs was shown and Flag tag was additionally added to the C terminus of the truncations. (**e**) 293T cells were transfected with the indicated truncated SARS-CoV-2 S2 constructs. Then, mAbs of B10, C10, E2, G5 and anti-Flag were respectively used as the primary antibody to perform western blot. **f,** The purified and unpurified ascites of B10, C10, E2 and G5 were analyzed by SDS-PAGE.


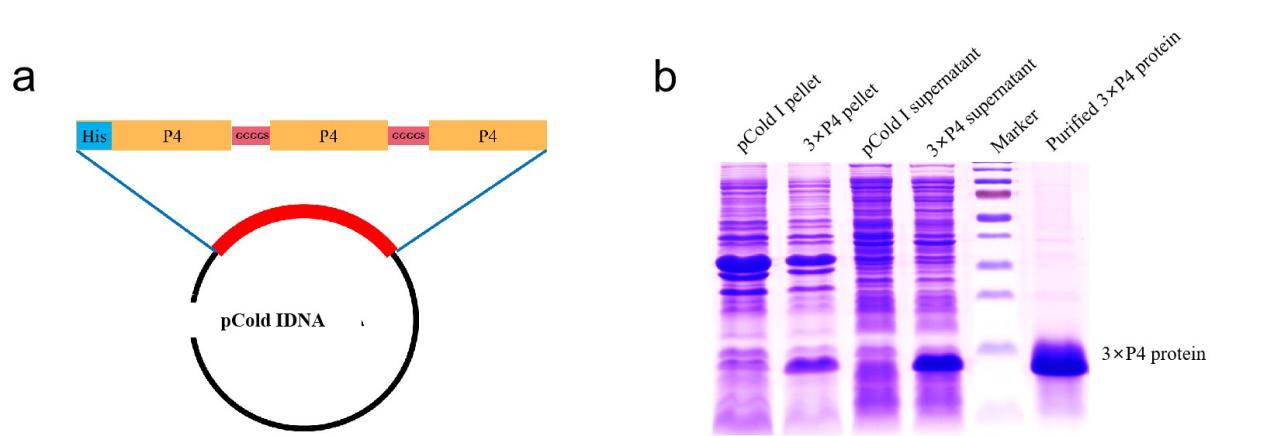


**Fig S2. Construction and expression of the trimeric P4 recombinant plasmid.**

**a,** The strategy for construction of the trimeric P4 expression recombinant plasmid. The three P4 fragments were amplified by PCR and every two P4 fragment were connected by GGGGS-linker. The three fragments were insert into pColdⅠ vector by homologous recombination technique and the generated plasmid was designated as 3×P4. **b,** The expression of the trimeric P4 protein. The 3×P4 and pColdⅠ vector were respectively transformed into BL21 (DE3) E.coli competent cells, and then the expression of the trimeric P4 protein was induced by IPTG and analyzed by SDS-PAGE.
